# Case report: Primary myelofibrosis presenting with portal hypertension mimicking cirrhosis

**DOI:** 10.3389/fmed.2024.1375571

**Published:** 2024-05-03

**Authors:** Xiayu Gan, Shengjie Yu, Min Zhu, Bo Ning, Song He, Xiaoyue Xie, Linshuang Tu, Huihong Yu

**Affiliations:** ^1^Department of Gastroenterology, The Second Affiliated Hospital of Chongqing Medical University, Chongqing, China; ^2^Department of Urology, The Second Affiliated Hospital of Chongqing Medical University, Chongqing, China; ^3^Department of Geriatrics, The First People’s Hospital of Neijiang, Neijiang, China

**Keywords:** primary myelofibrosis, portal hypertension, cirrhosis, liver biopsy, treatment

## Abstract

Primary myelofibrosis (PMF) is an infrequent etiology of noncirrhotic portal hypertension (PH). In clinical settings, non-cirrhotic PH is often misdiagnosed as cirrhotic PH. This case report details a patient who exhibited recurrent esophageal variceal hemorrhage and was initially misdiagnosed with cirrhosis. Initially poised for liver transplantation, the patient’s liver biopsy revealed no significant cirrhosis but showed signs of extramedullary hematopoiesis (EMH). Following the accurate diagnosis of PMF, the patient underwent standard treatment, leading to an absence of recurrent gastrointestinal hemorrhage due to esophageal varices for nearly three years.

## Introduction

Primary myelofibrosis (PMF) is a myeloproliferative neoplasm characterized by a wide spectrum of clinical manifestations (1). It stands as an uncommon etiology of non-cirrhotic portal hypertension (PH) (2), where the initial presentations in PMF patients with PH may emerge as complications of the PH itself. Consequently, distinguishing these patients from those with cirrhosis poses a significant challenge. Herein, we present the case of an elderly male who, in anticipation of liver transplantation due to recurrent esophageal variceal hemorrhage, was ultimately diagnosed with PMF following liver biopsy, bone marrow examination, and genetic analysis. This case prompts a review of existing literature on PMF associated with PH. While the majority of reported cases identify PH subsequent to a PMF diagnosis, the patient in this case exhibited PH prior to the identification of PMF.

### Case presentation

A 64-year-old Asian male was admitted to the hospital on October 11, 2021, for preoperative preparation for a liver transplantation intended to address decompensated cirrhosis. The patient had a long history of complications related to cirrhosis. Notably, the patient had no family or personal history of other significant illnesses. His treatment history was primarily focused on symptomatic management of cirrhosis complications. His medical history included episodes of ascites and melena, alongside symptoms of splenomegaly and anemia, though his white blood cell and platelet counts remained within normal ranges. Due to gastrointestinal hemorrhage, he underwent esophageal variceal ligation on two occasions. Physical examination at admission noted a slight jaundice indicated by yellowish sclera, but no ecchymosis or petechiae were observed on the skin across his body. The cardiopulmonary examination was unremarkable. Abdominal examination revealed palpable splenomegaly with the spleen’s margin extending beyond the ribcage, reaching above the umbilical level and the anterior median line, without any other notable abdominal findings.

Upon admission, the patient exhibited a peripheral blood erythrocyte count of 3.78 × 10^12^/L, which is below the normal range of 4.30–5.80 × 10^12^/L. Hemoglobin levels were measured at 90 g/L, also falling short of the reference range (130–175 g/L). Counts for white blood cells, neutrophils, lymphocytes, and platelets remained within expected limits. Liver function tests revealed elevated total bilirubin at 42.8 μmol/L (normal range: 5.1–28.0 μmol/L), direct bilirubin at 21.5 μmol/L (normal range: 0.0–10.0 μmol/L), indirect bilirubin at 21.3 μmol/L (normal range: 1.5–18.0 μmol/L), and total bile acids at 21.6 μmol/L (normal range: 0.0–10.0 μmol/L), whereas levels of aminotransferases, γ-GGT, and alkaline phosphatase (ALP) were found to be within the normal range. Analysis of digestive tumor markers indicated a CA-125 level of 109.0 μmol/L, significantly above the reference range (0.0–35.0 μmol/L), while levels of AFP, CEA, and CA-199 were found to be within normal parameters. Immunological assessments showed increased levels of IgG at 17.7 g/L (normal range: 7.00–16.0 g/L), IgM at 4.09 g/L (normal range: 0.4–2.3 g/L), and IgA at 5.71 g/L (normal range: 0.7–4.0 g/L), alongside a positive ANA result at 1:100 with cytosolic granules. Tests for ALMA, ASMA, LKM, SLA, AMA, and AMA-M2, including the IgG4 subtype of immunoglobulin, were negative. No significant abnormalities were detected in coagulation profiles, electrolyte levels, renal function, ceruloplasmin levels, or viral hepatitis markers. The pivotal laboratory findings are summarized in Table 1.

**Table 1:** Significant laboratory results of the patient.

Laboratory examination	Results	Reference range
Red Blood cell count (×10^12^/L)	3.78	4.30–5.80
Hemoglobin (g/L)	90	130–175
Mean Corpuscular Volume (MCV) (fL)	82.0	82.0–100.0
White Blood Cell (WBC) Count (×10^9^/L)	3.82	3.50–9.50
Neutrophil Percentage (%)	84.6	45.0–75.0
Neutrophil Count (×10^9^/L)	3.23	1.80–6.30
Lymphocyte Percentage (%)	8.8	20.0–50.0
Lymphocyte Count (×10^9^/L)	0.34	1.10–3.20
Monocyte Percentage (%)	1.8	3.0–10.0
Monocyte Count (×10^9^/L)	0.07	0.10–0.60
Eosinophil Percentage (%)	4.4	0.4–8.0
Eosinophil Count (×10^9^/L)	0.17	0.02–0.52
Basophils Percentage (%)	0.4	0.0–1.0
Basophils Count (×10^9^/L)	0.01	0.00–0.06
Platelet Count (×10^9^/L)	104	100–300
Alanine Aminotransferase (ALT) (U/L)	9	9–50
Aspartate Aminotransferase (AST) (U/L)	24	15–40
Alkaline Phosphatase (ALP) (U/L)	93	45–125
γ-Glutamyl Transferase (γ-GGT) (U/L)	29	10–60
Total Bile Acids (umol/L)	21.6	0.0–10.0
Total Bilirubin (umol/L)	42.8	5.1–28.0
Direct Bilirubin (umol/L)	21.5	0.0–10.0
Albumin (g/L)	34.8	40.0–55.0
Prothrombin Time (PT) (s)	16.8	11.0–14.5
Erythropoietin (EPO) (mIU/mL)	12.24	2.59–18.50
Uric Acid (umol/L)	466.0	210.0–430.0
Lactate Dehydrogenase(LDH) (U/L)	307	120–250
Ferritin (ng/mL)	27.60	23.90–336.20

**Figure 1:**
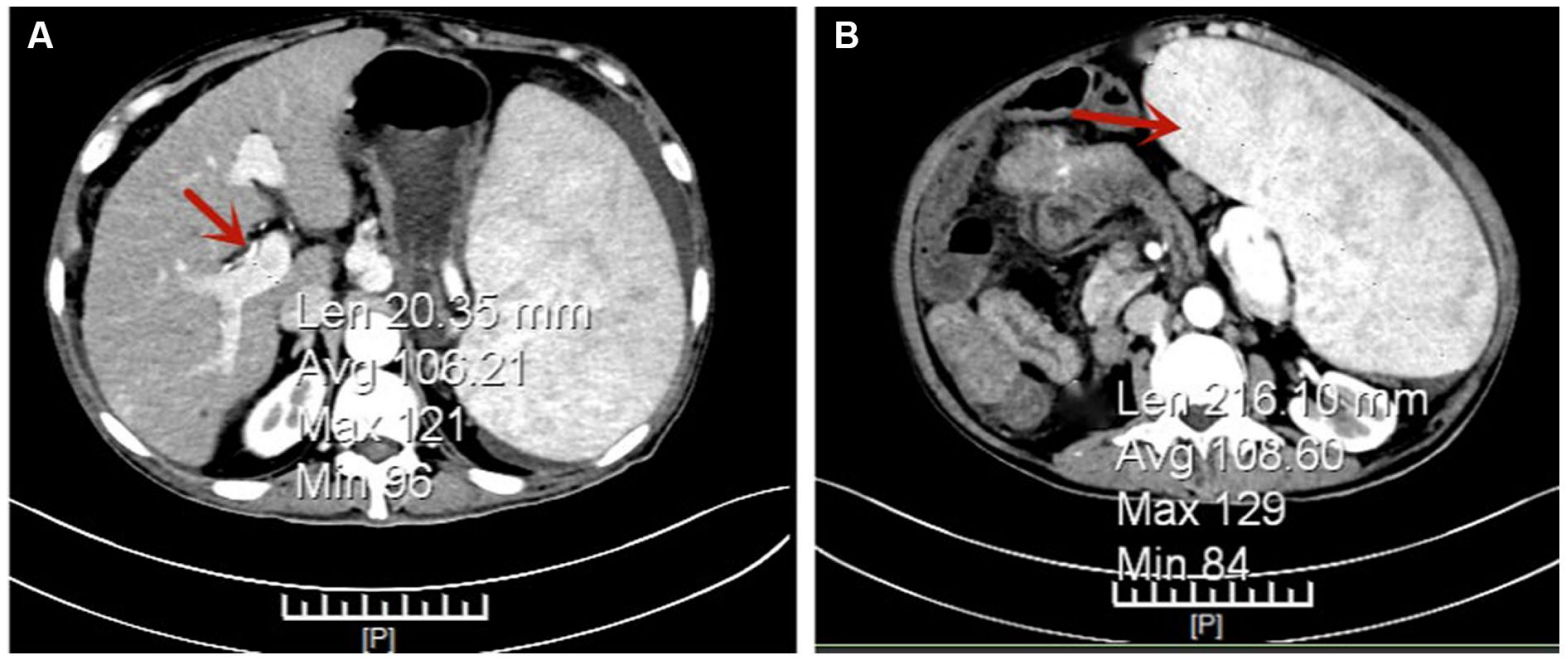
Portal Vein Computed Tomography Venography (CTV) Indicating Pulmonary Hypertension (PH). Panel **(A)** shows the main portal vein with a width of approximately 20 mm, as highlighted by the arrow. Panel **(B)** demonstrates a significantly enlarged spleen, as indicated by the arrow.

**Figure 2:**
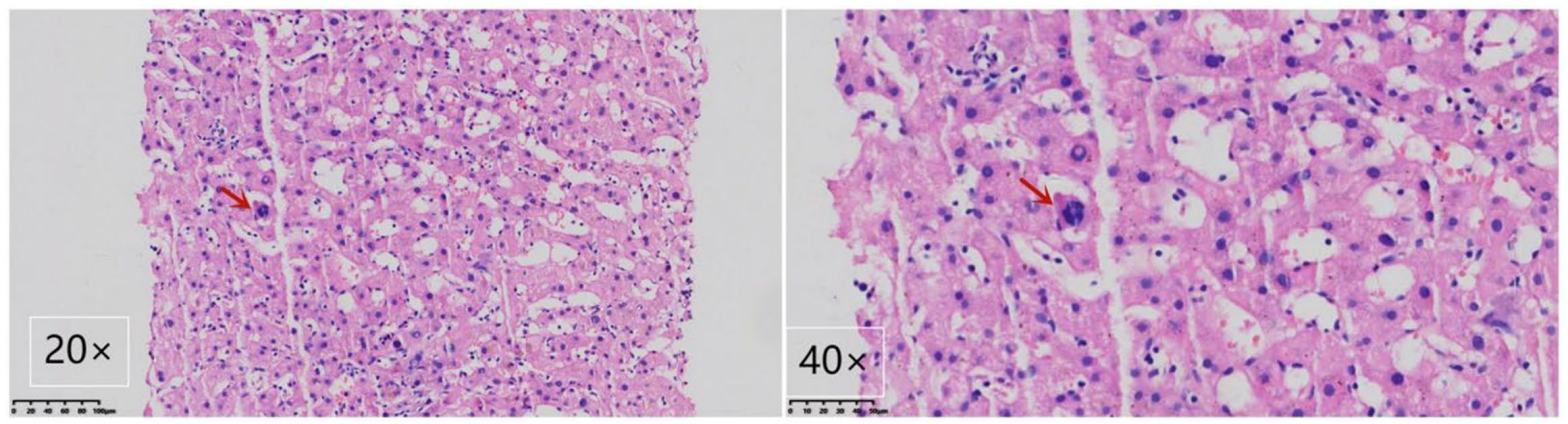
Liver biopsy demonstrating myeloid metaplasia in the patient. A megakaryocyte is highlighted by the arrow, showcasing the presence of myeloid metaplasia. The image includes views at magnifications of 400× and 200× using Hematoxylin-Eosin staining.

**Figure 3:**
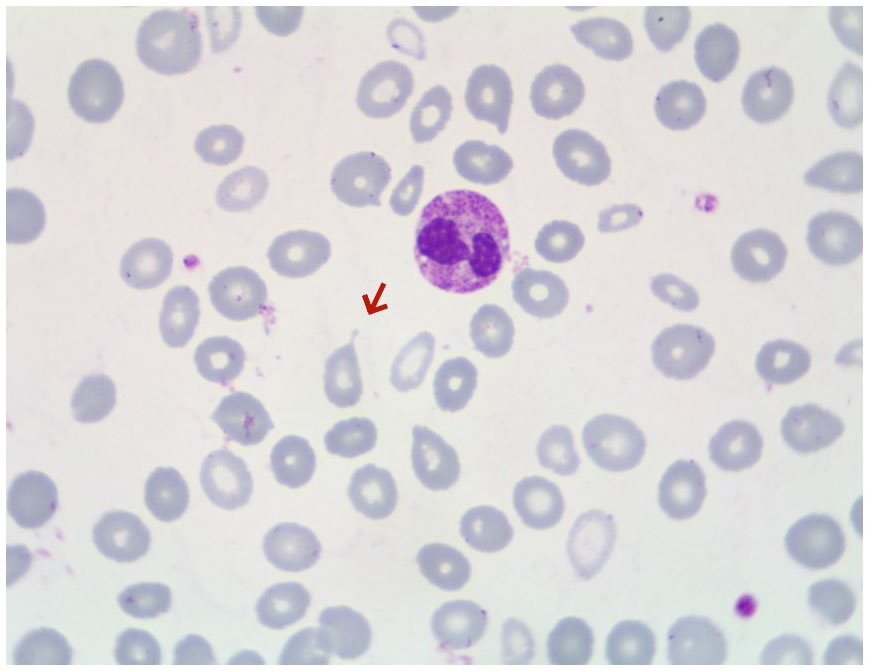
Peripheral blood smear of the patient, with arrows indicating teardrop-shaped red blood cells.

**Figure 4:**
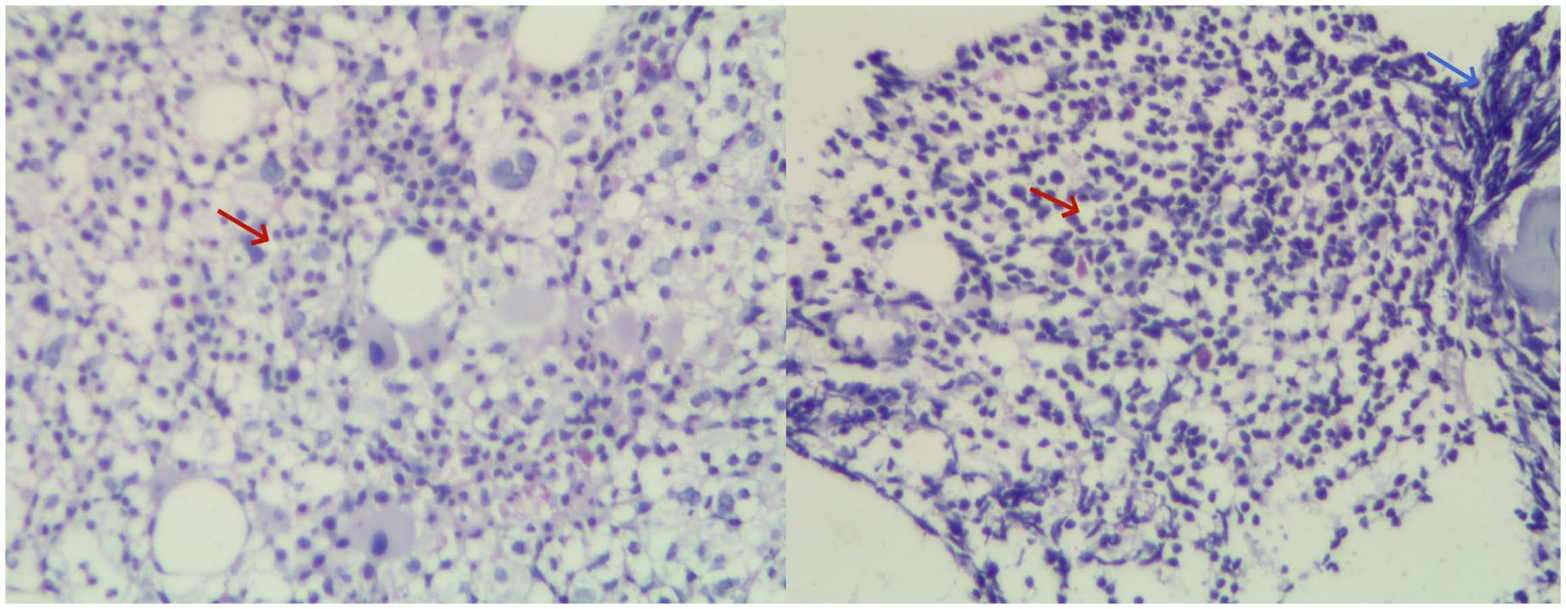
Combined image of hematoxylin and eosin (HE) staining of bone marrow biopsy at 20× magnification. Clusters of megakaryocytes are indicated by red arrows, while bone marrow fibrosis is highlighted by blue arrows.

Gastroscopy revealed the presence of 3–4 varicose veins within the esophagus, each displaying red signs indicative of potential bleeding risks. Computed Tomography Venography (CTV) of the portal vein highlighted irregular liver contours, splenomegaly, and evidence of pulmonary hypertension, as illustrated in Figure 1. Duplex ultrasonography of the portal vein detected no signs of portal vein thrombosis and measured the main portal vein’s diameter at approximately 19 mm. A liver biopsy demonstrated sinusoidal hematopoiesis without the presence of cirrhosis, depicted in Figure 2. Given the absence of cirrhosis in the liver histological findings and the blood tests indicating anemia without leukopenia or thrombocytopenia, the patient was recommended to undergo further examinations for potential hematological disorders. The peripheral blood smear of the patient reveals teardrop-shaped red blood cells with no apparent leukoerythroblastic features, as depicted in Figure 3. The bone marrow biopsy results of the patient revealed extensive proliferation of marrow fibrous tissue, with a proliferation rate exceeding 90%, stained with HE and PAS. There was a reduction in the granulocyte-erythrocyte ratio, with various stages of granulocytic cells observed, predominantly consisting of cells at the myelocyte stage or earlier. Various stages of erythroid cells were also visible, predominantly comprising cells at the metamyelocyte and later stages. Megakaryocytes were readily observed, scattered or clustered, with megakaryocytes showing nuclear hyperchromasia and clustering, as depicted in Figure 4. The reticulin stain suggests MF-2 grade. Genetic testing identified the JAK2 V617F mutation, a gene associated with Primary Myelofibrosis (PMF). Absence of significant symptoms such as erythrocytosis, thrombocytosis, headache, vision loss, or pruritus alongside the bone marrow biopsy indicating significant fibrosis led to the diagnosis of PMF, rather than Polycythemia Vera (PV) or Essential Thrombocythemia (ET). The patient received treatment with ruxolitinib in the Hematology Department, successfully avoiding gastrointestinal bleeding and ascites development.

## Discussion

In examining the prevalence of pulmonary hypertension (PH) in patients with Primary Myelofibrosis (PMF), a previous study identified a prevalence rate of 7.38% (3), suggesting that PH is a relatively common complication in individuals with PMF. This condition often manifests with symptoms akin to those observed in decompensated cirrhosis, including esophageal varices and ascites.

Individuals diagnosed with Primary Myelofibrosis (PMF) commonly exhibit nonspecific symptoms initially, including fatigue, night sweats, episodes of angina, and significant weight loss, before any symptoms associated with pulmonary hypertension (PH) emerge. Additionally, a marked elevation in white blood cell count (WBC > 11 × 10^9^/L) serves as a secondary diagnostic indicator for PMF (4), reflecting the abnormal clonal proliferation occurring within bone marrow hematopoiesis. The case in question, however, presents an atypical manifestation, predominantly displaying symptoms characteristic of PH, such as ascites and variceal bleeding, in conjunction with slightly abnormal liver function tests. The patient’s ongoing moderate anemia was preliminarily ascribed to gastrointestinal hemorrhage. This deviation from the expected clinical presentation complicates the differential diagnosis process, particularly in distinguishing between PMF and decompensated cirrhosis. The overlapping symptomatology underscores the diagnostic challenges faced in these scenarios, emphasizing the intricacies involved in accurately diagnosing conditions with similar clinical manifestations.

The differential diagnosis of pulmonary hypertension (PH) encompasses conditions both with and without cirrhosis, where clinical differentiation largely relies on imaging studies and liver histology. The patient’s computed tomography (CT) scan may have led to a preliminary misdiagnosis of cirrhosis, attributed to the liver’s uneven edges—a feature potentially caused by myeloid metaplasia, echoing the misinterpretation seen in imaging in a previously documented case (5). Due to the invasive nature of liver biopsy, it was not conducted during the initial admission. The diagnosis of Primary Myelofibrosis (PMF) is contingent upon bone marrow examination and genetic analysis. Initial findings of anemia were attributed to bleeding from esophageal varices, which delayed the consideration for bone marrow aspiration.

Notably, the patient exhibited significant splenomegaly, with ultrasound measurements of the spleen reaching 26.0 × 8.9 cm. Despite the presence of anemia, there was no observation of leukopenia or thrombocytopenia, symptoms that could not be accounted for by hypersplenism alone. These clinical presentations do not align with a cirrhosis diagnosis. Consequently, a liver biopsy was recommended and subsequently performed, revealing no significant evidence of cirrhosis but confirming extramedullary hematopoiesis (EMH), as depicted in Figure 2. It was then advised that the patient undergo a bone marrow biopsy and genetic testing. Following these procedures, a final diagnosis of PMF was established, underscoring the importance of comprehensive diagnostic evaluations in distinguishing between PH due to PMF and cirrhosis, particularly when initial symptoms and imaging findings may lead to potential diagnostic ambiguities.

This case underscores the critical role of liver biopsy in accurately differentiating between pulmonary hypertension (PH) caused by Primary Myelofibrosis (PMF) and cirrhosis. In patients with myelofibrosis, PH is commonly associated with portal or hepatic vein thrombosis (6). However, in scenarios where no such thrombosis is observed, the development of PH could be linked to an elevation in intrahepatic resistance, which may arise from extramedullary hematopoiesis (EMH), or due to a significant increase in portal blood flow as a consequence of splenomegaly (7–9). EMH stands out as a pivotal pathological process in the onset of PH, with 90–100% of PMF patients exhibiting varying degrees of EMH in the liver (10).

In the discussed case, computed tomography venography (CTV) of the portal vein and color Doppler ultrasound of the hepatic vein did not reveal any thrombi or significant thickening of the splenic vein, while a liver biopsy confirmed the presence of sinusoidal hematopoiesis. The patient’s condition limited the assessment of portal vein blood flow and velocity through Doppler ultrasound, and hepatic venous pressure gradient (HVPG) measurements were not conducted. Nonetheless, the inner diameter of the main portal vein, approximately 19 mm as determined by ultrasonography, indicates potential alterations in portal circulation. Consequently, it is inferred that the patient’s PH may stem from sinusoidal obstruction due to EMH coupled with an enhanced blood flow in the splenic vein, a result of splenomegaly. This analysis highlights the intricate relationship between PMF-associated pathologies and the development of PH, accentuating the need for thorough diagnostic processes to inform effective treatment strategies.

The management strategies for portal hypertension (PH) secondary to primary myelofibrosis (PMF) remain an area of ongoing research and development. In our literature review, several interventions have been proposed, including the administration of ruxolitinib, endosonographic coiling, targeted application of cyanoacrylate, endoscopic variceal ligation (EVL), endoscopic injection sclerotherapy (EIS), among other approaches (5, 9–19). EVL and EIS, in particular, have demonstrated efficacy in managing esophageal variceal bleeding attributable to PH in PMF patients. The transjugular intrahepatic portosystemic shunt (TIPS) procedure emerges as a viable option to reduce intrahepatic resistance, especially following the unsuccessful application of conservative and endoscopic treatments.

In the case examined, the patient underwent two endoscopic variceal ligations and subsequently remained free from upper gastrointestinal bleeding for a duration of two years. This outcome not only highlights the effectiveness of endoscopic variceal ligation in the treatment of variceal bleeding in PMF patients with PH but also supports the broader feasibility of endoscopic interventions in such clinical scenarios.

## Data availability statement

The original contributions presented in the study are included in the article/supplementary material, further inquiries can be directed to the corresponding author.

## Ethics statement

Written informed consent was obtained from the individual(s) for the publication of any potentially identifiable images or data included in this article.

## Author contributions

XG: Conceptualization, Data curation, Formal analysis, Funding acquisition, Investigation, Methodology, Project administration, Resources, Software, Supervision, Validation, Visualization, Writing – original draft, Writing – review & editing. SY: Writing – original draft, Writing – review & editing. MZ: Writing – original draft, Writing – review & editing. BN: Writing – original draft, Writing – review & editing. SH: Writing – original draft, Writing – review & editing. XX: Writing – original draft, Writing – review & editing. LT: Writing – original draft, Writing – review & editing. HY: Writing – original draft, Writing – review & editing.
